# Curve-fitting techniques improve the mid-infrared analysis of soil organic carbon: a case study for Brookston clay loam particle-size fractions

**DOI:** 10.1038/s41598-018-30704-2

**Published:** 2018-08-15

**Authors:** Ruqin Fan, Xueming Yang, Craig F. Drury, Zhenhua Zhang

**Affiliations:** 1 Institute of Agricultural Resources and Environment, Jiangsu Academy of Agricultural Sciences, Nanjing, 210014 China; 2 Harrow Research and Development Centre, Agriculture and Agri-Food Canada, Ontario, N0R 1G0 Canada

## Abstract

Few studies have evaluated structural features of soil organic carbon (SOC) in different soil particle fractions, especially SOC changes induced by tillage, using Fourier transformed mid-infrared (MIR) spectroscopy. To make a contribution in this context, soil samples of a Brookston clay loam (mesic Typic Argiaquoll) with averaged pH and organic matter concentration at 7.28 and 43.9 g kg^−1^, respectively, were collected from short-term no-tillage (NT97) and mouldboard plow (CT97) treatments initiated in 1997 and long-term no-tillage (NT83) and mouldboard plow (CT83) treatments initiated in 1983 under a corn-soybean rotation, and were separated into sand, silt, and clay fractions using sonication. Structural features of SOC in these soil fractions were investigated using curve-fitting analysis of mid-infrared (MIR) spectra. Aromatic C content was found to be greater in clay- than in sand-sized fractions, while aliphatic C content was higher in sand- than in silt- and clay-sized particles. With decrease in tillage intensity, the aliphatic C gradually increased in sand- and clay-sized fractions but not in the silt-sized fraction. The aliphatic C content in sand fraction was significantly greater in NT83 than CT83 (*P* < 0.05). The aromatic C in silt- and clay-sized fractions was greater in NT83 than in both CT soils, whereas aromatic C contents were higher in both CT soils than in NT83 soil. Significantly higher aromatic/aliphatic C ratio in CT83 than NT83 was found in sand-sized fractions, while the opposite trend was found in the silt-sized fraction. These findings were not apparent until the curve-fitting technique was employed, which has the capacity to quantify many overlapped bands in the spectra. This study demonstrates that the curve-fitting of MIR spectra advances the analysis of organic matter in soil samples.

## Introduction

Soil organic carbon (SOC) content and quality were found to vary with tillage systems and soil depth^1–3^, with crop rotation^4^, as well as with particle size fractions^5,6^. Characterization of SOC chemical composition in different soil size fractions (e.g., sand, silt and clay) has shown that different-sized minerals potentially protect different types of SOC, thus physical fractionation of primary organo-mineral complexes provides a useful approach to understand the dynamics and functional attributes of SOC^7,8^. Moreover, the SOC in physical size fractions responds differently to soil cultivation and management changes^9^. The content of SOC in sand sized fractions is enriched in plant residues, while the SOC associated with silt particles is enriched in microbially-transformed plant residues^10^. Soil organic C has been found to be significantly correlated to clay content in numerous studies^11,12^.

The ^13^C NMR spectroscopy analysis is a powerful means for studying the SOC characteristics associated with soil particle-size fractions^10,13^. Despite its efficacy, NMR technique needs significant economic resources, material, equipment and time^14^. Other techniques have also been used to study SOC properties, such as pyrolysis-field ionization mass spectrometry. Using this technique, the abundance of carbohydrates, phenols and lignin monomers, alkylaromatics and N-containing compounds was found be vary distinctly among the physical fractions^15^. It was found using laser induced fluorescence analysis that the SOC was less humified under NT than under other tillage practices and the SOC associated with clay fraction in particular was less humified than associated with other particle size fractions of an Oxisol^16^. Infrared (IR) spectroscopy is used to identify the chemical compounds and their specific functional groups for many organic materials including soils^17^. In particular, mid-IR (MIR) is becoming increasingly common due to the specificity of the absorption bands. Different intensities of carboxylic, aromatic, CH and NH groups in a variety of bulk soils and silt fractions with different fertilizer amendments were reported, and these findings were based on relative absorbance of distinct peaks^18,19^. Calderón *et al*.^20^ investigated the changes of SOC chemistry in different SOC fractions during incubation using MIR spectroscopy. The variation of soil organic matter quality among differently managed sites was detected using MIR spectroscopy^21^. However, limited work has been done on differentiating the characteristics of SOC in soil particle size fractions using MIR spectroscopy, particularly with soils under different tillage systems. Furthermore, most studies were completed using the original MIR spectra where important absorption information may be missing in the overlapping bands due to the unavoidable interferences with minerals. Overlapping bands make it difficult to directly evaluate parameters such as position, width and area^22^. To resolve this problem, a curve-fitting technique, mainly based on Levenberg-Marquardt algorithm^23,24^, has been proposed as a useful and robust method to estimate these band parameters due to its potential in resolving overlapped bands into distinct peaks^25–27^. It has been reported that the curve fitting method provided more feasible results for the band shifts than the second derivative method in the analysis of overlapping overtones^25^. The principle behind the curve-fitting technique is to represent peaks using analytical functions with some undetermined parameters and optimize these parameters to approximate the actual curve^28^. Lin *et al*.^29^ successfully discriminated the compositions in loratadine/hydroxypropyl-ß-cyclodextrin inclusion complex using curve-fitting analysis of Fourier-transform infrared (FTIR) spectroscopy. Other studies have also validated the curve-fitting method in medical science^30^ and optical engineering applications^31^. O’Shaughnessy *et al*.^32^ revealed properties of caesium silicate glasses at different temperature by identifying curve fitted bands based upon Raman spectroscopy. Katsumi *et al*.^33^ revealed properties of humic substances from Japanese Andisols, Inceptisols and Entisols by identifying curve fitted bands based upon X-ray diffraction spectroscopy. Nevertheless, there is a lack of information using the curve-fitting techniques for SOC analysis, especially for tillage induced SOC changes. We hypothesize that curve-fitting of MIR spectrum of soils will reveal significant more SOC information than the MIR spectrum itself. The objectives of this study were to (i) use curve-fitting to identify functional groups from the MIR spectra of soil samples which have distinct overlapping bands and (ii) assess whether curve-fitting can reveal the impacts of tillage practices on structural features of SOC in light of our current understanding of SOC associated with physical-size fractions in soil.

## Materials and Methods

### Site description and soil sampling

Soil samples were collected from the 0–10 cm layer of a tillage trial conducted on Brookston clay loam soil (poorly drained; lacustrine; mixed, mesic Typic Argiaquoll) at the Eugene Whelan Research Farm, Agriculture and Agri-Food Canada, Woodslee, Ontario (42°13′N, 82°44′W). The mean annual air temperature at the study site is 8.9 °C and the mean annual precipitation is 832 mm. The average texture in the top 10 cm was 258 g kg^−1^ sand, 336 g kg^−1^ silt, and 406 g kg^−1^ clay. The averaged soil pH and soil organic matter content was 7.28 and 43.9 g kg^−1^, respectively. Five treatments were involved in this study, including (1) a long-term no-tillage established in 1983 (NT83), (2) a long-term conventional tillage established in 1983 (CT83), (3) a short-term no-tillage established in 1997 (NT97) after 13 years of CT, (4) a short-term conventional tillage established in 1997 (CT97) after 13 years of NT, and (5) a long-term Kentucky bluegrass SOD established in 1983 (SOD83). All tillage treatments were under a corn-soybean rotation and soil samples were collected from the corn phase. All treatments were arranged in complete randomized block design with two field replicates.

### Soil particle fractionation

Soil samples were separated into particle size fractions, including sand- (2000-53 μm), silt- (53-2 μm), and clay- (<2 μm) size fractions (organic-mineral complexes) using a sonication procedure as described in a previous study^34^ in 4 replications. Briefly, 20 g of bulk soil (<2 mm) were placed in a 250 mL breaker with 80 mL of distilled water. The soil-water suspensions were treated with ultrasound using an energy level of 750 J mL^−1^ and were then washed through a 53 μm sieve with distilled water into a 2 L cylinder. The sand-size fraction was washed and transferred into an aluminium container, and then oven-dried at 55 °C. The clay was separated from the silt by repeated gravity sedimentation^35^, and then the clay and silt fractions were concentrated using centrifugation. The clay and silt pellets were treated by the method of Gregorich *et al*.^36^. The clay, silt and sand fractions refer to clay-, silt- and sand-sized organic-mineral complexes in this study. The SOC concentrations of the bulk soil and particle size fractions were determined using a Leco CNS 2000 analyzer (Leco Cor., St. Joseph, MI) (Table 1). The surface soils were free of carbonates, so total C was equivalent to organic carbon as reported by Zhang *et al*.^34^. The samples of sand size fractions were ground with a mortar and pestle and stored for MIR spectra collection.

**Table 1:** The concentrations of soil organic carbon in whole soil and soil particle size fractions (0–10 cm).

Treatments^†^	Whole soil	Sand (53–2000 µm)	Silt (2–53 µm)	Clay (<2 µm)
	g C kg^−1^ sample			
SOD83	52.6 ± 5.01	18.8 ± 1.69	39.8 ± 4.17	44.3 ± 3.76
CT83	24.4 ± 2.39	4.1 ± 0.24	21.1 ± 3.08	28.5 ± 3.02
CT97	21.4 ± 3.20	5.7 ± 0.31	24.8 ± 1.99	32.7 ± 2.73
NT83	29.1 ± 1.93	7.4 ± 0.59	25.0 ± 2.47	34.6 ± 2.49
NT97	27.1 ± 2.04	6.8 ± 0.33	23.2 ± 2.05	33.8 ± 3.18

Values are means of four replicates.

^†^SOD83, long-term Kentucky bluegrass established in 1983; CT83, long-term conventional tillage established in 1983; CT97, short-term conventional tillage established in 1997 following 13 years of no-tillage (NT); NT83, long-term NT established in 1983; NT97, short-term NT established in 1997 following 13 years of CT.

### Fourier-transform diffuse reflectance MIR spectroscopic analysis

About 100 mg of each sample, including clay-, silt- and sand-sized fraction, were scooped into a stainless-steel sample cup, levelled with a spatula and placed on a Pike EasyDiff assembly (Easidiff, Pike Technologies, Madison, WI) on a Bruker-TENSOR 37 spectrometer (Bruker Optik GmbH, Ettlingen, Germany) for diffuse reflectance Fourier Transform MIR spectral collection. Three separate MIR spectra for each sample were obtained from three subsamples in the range of 4000 to 500 cm^−1^ with 64 scans and 2 cm^−1^ resolution. The background spectrum was determined using potassium bromide (KBr)^37^. Spectra data were pre-processed and evaluated with the OPUS-6.5 software (Bruker Optik GmbH, Germany) using spectra averaging, baseline correction and peak picking techniques.

**Figure 1:**
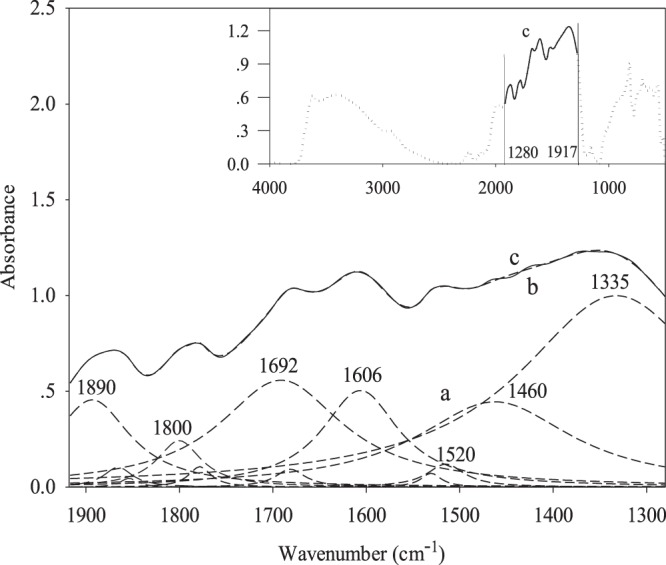
An example of curve-fitting analysis of mid-infrared spectrum (SOD soil). (**a**) Deconvoluted spectra resolved from curve-fitting, (**b**) fitted spectrum, and (**c**) the original spectrum. Insert: curve-fitting process applied to the range of 1280–1917 cm^−1^.

Curve-fitting was performed using the OPUS-6.5 Software to handle the complex spectra of overlapped bands on the basis of the second derivative and Levenberg-Marquardt algorithm and the Lorentz method for the peak shape. The curve-fitting is also known as band-fitting or non-linear regression. It is a mathematical tool for modeling the experimental data. The model adjustable parameters, contained in the vector α of length M, are varied until the best agreement between data and model is obtained. The least square solution is considered to be the best under most conditions obtained by minimizing the objective function $\sum _{i=1}^{N}\,{(y(vi)-f(vi;\alpha ))}^{2}$, where the experimental data as a function of the variable v is given by *y*(*vi*) and the number of experimental data by *N*. The fitted data is given by *f*(*vi*; *α*). The number of bands, their positions, intensities and widths were determined by fitting a highly deconvoluted spectrum. The deconvolution of spectra would allow the partial resolution of otherwise overlapped bands. The procedure of curve-fitting in this study was as follows^22^: (i) the frequency range of a spectrum were selected after baseline correction, (ii) overlapped peaks were identified and selected for analysis, (iii) curve-fitting of the deconvoluted spectrum to the original spectrum was performed using the curve fit module of the OPUS-6.5 Software. The standard error was controlled between 0.02–0.04 which indicates the accuracy and efficacy of the fitting process during calculation of single component of overlapped bands with the module. For all particle fractions under short- and long-term tillage managements as well as SOD83, the entire frequency range of 4000-500 cm^−1^ was deconvoluted and curve-fitted to evaluate the impacts of tillage practices on the compositions of SOC. The curve-fitting process in the frequency range from 1280–1917 cm^−1^ of the SOD83 soil was given as an example to illustrate the feasibility of this method for qualitative and quantitative study of SOC (Fig. 1). Infrared absorption bands of functional groups in soil samples were assigned following a literature review and the functional groups belonging to aliphatic and aromatic types were identified (Table 2). At identified peaks, upper and lower boundaries were established, after which a baseline was drawn between the boundaries and an integration was performed to calculate the corrected peak area. Peak area integration on the corrected spectra was then performed using the spectral processing software OPUS 6.5 (Bruker Optik GmbH)^38^. The intensity and integral of either aliphatic or aromatic group were the sum of the relative absorption intensity and peak area of single peaks in either group for a given sample and were used as relative quantity of different functional groups. Four replicates were conducted for spectra collection and analysis.

**Table 2:** Absorption bands in MIR spectra and assignments.

Wavenumber (cm^−1^)	Assignments	Cited references
775, 830^‡^	Aromatic C-H out of plane bending	^67^
1000–1080^†^	C-O stretching of cellulose	^68^
950–1170^†^	Polysaccharides or polysaccharides-like substances	^40, 69^
1200–1280	C-O stretching and OH deformation of COOH, C-O stretching of aryl ethers	^40^
1350^†^	COO- stretching and/or –CH bending of aliphatics	^67^
1450–1460^†^	Aliphatic C-H	^40^
1500–1510^‡^; 1525^‡^	Aromatic C=C and C-H stretching	^67, 70^
1565	Carboxyl C	^69^
1600–1620	Aromatic C=C and/or –COO streching	^40^
1690–1730; 1720–1725	C=O stretching of –COOH and ketones	^40, 71^
1790–1990	Quartz	^39^
2600	O-H stretching of H-bonded -COOH	^67^
2850–2920^†^	Aliphatic C-H	^70^
2869^†^	H-C-H asymmetric & symmetric stretching	^72^
2940^†^	Aliphatic C-H stretching	^40^
3030^‡^	Aromatic C-H stretching	^67^
3076^‡^	C=C-H stretching of aromatic rings	^72^
3204^†^	≡C-H stretching of alkynes	^73^
3300–3400	N-H, O-H stretching (H bonded groups)	^40^
3634	O-H stretching of clay and Fe oxides	^74^
3600–3700	OH of clay and Fe oxides	^74^

^†^Indicates aliphatic carbon; ^‡^indicates aromatic carbon.

### Statistical analysis

Analysis of variance (ANOVA) was conducted on the relative intensity and integral of different functional groups in particle size samples of soils under the four tillage managements as well as SOD83 before and after curve-fitting using SPSS 13.0 software package. The ANOVA was performed on the distribution of aliphatic and aromatic C in soil particle size fractions for each tillage treatments. When ANOVA was significant, the comparisons of aliphatic or aromatic C content among particle fractions and among tillage systems were examined using least significant difference test (LSD) at the 5% probability level.

## Results and Discussion

### Absorption bands of MIR spectrum in different particle size samples

**Figure 2:**
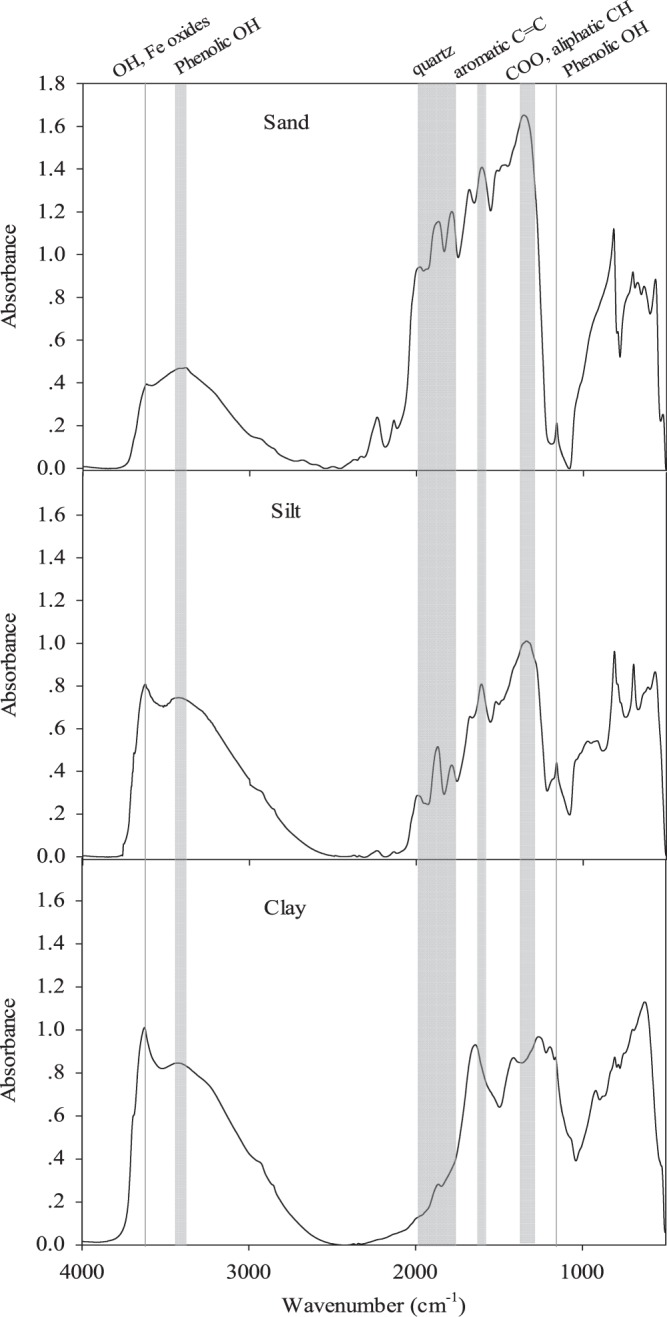
MIR spectra of different particle size fractions from a Brookston clay loam (0–10 cm) after 26 years of continuous Kentucky bluegrass management (SOD83) in Southwestern Ontario, Canada.

There were noticeable differences in the MIR spectra between the particle size fractions (Fig. 2). Most of the major absorption bands found in this study could be interpreted using published results^39–43^. The spectrum of the clay samples generally showed less distinct absorption bands compared with other particle fractions. The sharp peak at around 3625 cm^−1^ could be due to the stretching of -OH and Fe oxides in soil minerals, and the broad band at around 3380 cm^−1^ is due to chemically combined water in the clay, the stretching of phenolic OH groups, or the hydrogen–bonded OH. The intensity of these two bands was the strongest in clay fraction and the weakest in sand fraction as expected. The peaks between 1990 cm^−1^ and 1790 cm^−1^ were evident in the sand and silt fractions but not in the clay fraction, which is indicative of the presence of quartz in these soil fractions. The peaks between 1620 cm^−1^ and 1600 cm^−1^ are indicative of aromatic C=C stretching and/or asymmetric –COO stretching and these were most pronounced in the sand fraction. Similarly, the peak at 1350 cm^−1^ (symmetric –COO stretching and/or bending of aliphatics) was most intense in sand and silt fractions, but was not that intense in clay fraction. The peak at 1270 cm^−1^ (phenolic –OH) was noticeable in the spectrum clay sample, but missing in the spectra of the other particle size fractions. The peak at 1170 cm^−1^ (aliphatic -OH or polysaccharides stretching) was weak in sand fraction but intense in clay fraction, which indicates that the clay could have higher absorption capacity towards polysaccharides and other aliphatic –OH rich substance rich relative to the coarser size fractions.

### Curve-fitting analysis: SOC under long-term Kentucky Bluegrass management

The absorption bands in an original spectrum only indicate the existence of certain functional groups, such as aromatic rings, phenols, alcohols, etc., in the corresponding sample, but do not reveal meaningful quantitative information. This is mainly due to the many overlapped and unidentified absorption bands in the spectrum, as was evident in Fig. 1. To resolve this problem, curve-fitting techniques identified multiple peaks with relative peak intensity and peak area as was depicted between the 1280 cm^−1^ and 1917 cm^−1^ range in Fig. 1. The components under the overlapped bands include carboxylic acid, phenolic compounds, aliphatic –CH, aromatic C, etc. (Table 2). The relative intensities of the resolved peaks were considerably different from the original spectrum with overlapped bands (Fig. 1), indicating that curve-fitting technique enables quantitative assessment of functional groups of SOC. Using this technique, Calemma *et al*.^44^ resolved two bands from an FTIR spectrum of an oxidized coal sample into nine bands, and Rennert *et al*.^45^ quantitatively defined the relative quantity of functional groups in clay and clay plus silt samples in the frequency range of 1800-1200 cm^−1^.

Overlapped individual bands were resolved into distinct peaks by applying curve-fitting analysis to the entire MIR range (4000 to 500 cm^−1^) of the SOD83 sample spectra (Table 3), after which the relative intensity and area for each individual band were calculated. The integral (the area of absorption band) was calculated using both band intensity and width parameters and could thus provide additional information on the quantity of the components. The intensity and integral of aromatic C were highest in clay-sized fraction, with 1.3 and 2.4 times greater integral than in silt- and sand-sized fractions, respectively. The relatively low aromatic C content in coarse-sized fractions of sand was consistent with the findings that this fraction was generally enriched in light materials containing partly decomposed plant remnants with low recalcitrance^46^. The intensity of aliphatic C (normally classified as readily decomposable C) was highest in sand fraction. It is worth noting that the intensity and integral of aliphatic C were noticeably lower in silt than in other fractions and the integral was significantly lower than sand fraction (*P* < 0.05), indicating less readily decomposable C in silt-sized relative to in sand- even clay-sized fractions of Brookston clay loam soil. These results agree with Bayer *et al*.^47^ who found a higher concentration of semiquinone free radicals in fine-silt (20-2 μm) fraction using electron spin resonance spectroscopy which is indicative of a higher C recalcitrance in this fraction than in other fractions. The aliphatic C content in clay fraction was 34.7% higher than that in silt fraction, indicating that a portion of C associated with clay is not recalcitrant. This demonstrates that the relatively labile C is physically associated with mineral-particles in the clay fraction^48^, in addition to the recalcitrant C. This result was consistent with Kadono *et al*.^49^ who noted the readily decomposable nature of clay-associated SOC. Christensen^50^ and Zhang *et al*.^34^ also found a shorter half-life for the readily decomposable C in clay- relative to in silt-sized fraction, indicating that the readily decomposable C was more easily mineralized in clay- than in silt-sized fraction. The intensity of C connected with oxygen atoms, which is reflective of the oxidization of SOC, was 1.21 and 1.94 times higher in the clay- than in the silt- and the sand-sized fractions, respectively (*P* < 0.05; Table 3). This was consistent with the ranking of the aromatic C distribution among the fractions.

**Table 3:** Relative intensity and integral of different functional groups in particle size samples of SOD soil before and after curve-fitting.

	Fraction	Aliphatic C-H and C-OH		Aromatic C-H and C-OH		C connected to oxygen atoms	
		Relative Intensity	Integral	Relative Intensity	Integral	Relative Intensity	Integral
After curve-fitting	clay	1.30a^‡^	229ab	3.63a	1041a	3.26a	999a
	slit	1.07a	170b	3.20ab	787ab	2.69ab	650ab
	sand	1.77a	312a	1.94b	429b	1.70b	461b
Before curve-fitting	clay	0.09B	—^§^	1.23A	—	0.83A	—
	slit	1.17AB	—	1.63A	—	0.50A	—
	sand	1.69A	—	1.77A	—	0.65A	—

Values are means of four replicates.

^‡^Different uppercase or lowercase letters in the same column indicate significant difference at 0.05 probability level.

^§^There was no integral calculation before curve-fitting.

These results demonstrate that the curve-fitting technique for MIR spectra of soil particle size fractions can readily identify important spectral information which is not evident in the original MIR spectra. The structural characteristics of SOC revealed through the curve- fitting technique generally agree with the results obtained using other methods such as pyrolysis-field ionization mass spectrometry, ^13^C NMR spectroscopy, and electron spin resonance spectroscopy^47–52^.

### Tillage-induced changes in carbon functional groups in particle size fractions

**Figure 3:**
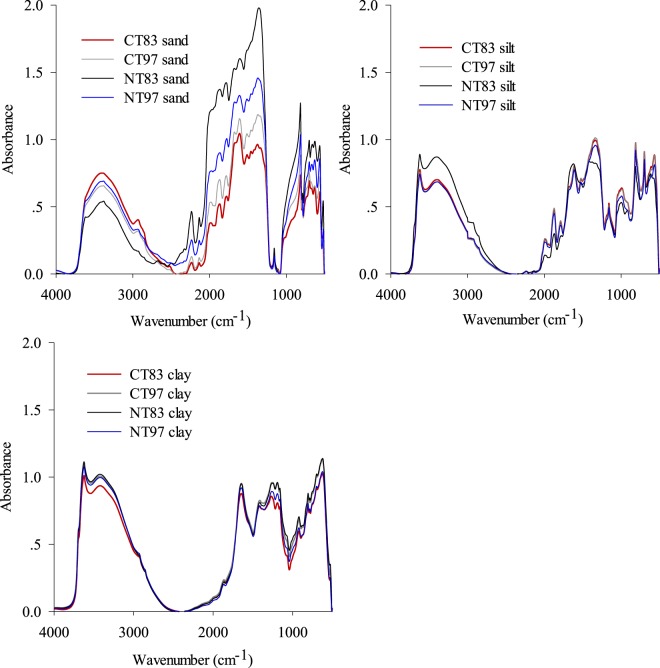
MIR spectra of soil particle size fractions from soils (0–10 cm) under different tillage practices. CT83 = long-term conventional tillage established in 1983; CT97 = short-term conventional tillage established in 1997 following 13 years of NT; NT83 = long-term no-tillage established in 1983; NT97 = short-term no-tillage established in 1997 following 13 years of CT.

Tillage treatments resulted in large differences in the MIR spectra of sand-sized fractions whereas only small differences were observed for the silt- and especially the clay-sized fractions (Fig. 3). The similar responses of sand MIR spectra to tillage changes was also observed in other North American soils^9,53^. This is consistent with the fact that the organic matter in the sand-sized fraction is the first pool that organic materials (typically plant residue and manure) enter into soil organic matter pool^54^. The coarse fraction of organic matter is composed of plant and fungal fragments and is considered to be the most sensitive pool which can reflect changes in soil organic matter due to changes in soil management practices^55,56^; while organic matter in fine-sized particles undergoes relatively slow changes during cultivation and contributes little to the short- and medium-term fertility of soils^57,58^.

Curve-fitting revealed that the aliphatic C in sand-sized fraction was also significantly higher under NT83 than under CT83 (Table 4). Aliphatic C content in the sand fraction was generally lower in the CT97 and CT83 than in NT97 and NT83 soils, indicating that the aliphatic C tends to be mineralized in conventional managed soils more so than in conservation managed soils. The integral of the aromatic C in the sand-sized fraction was 7.0%, 14.3% and 15.5% greater under CT83 than under CT97, NT97 and NT83, respectively. As a result, aromatic to aliphatic C ratio in sand-sized fraction, which in some degree reflects SOC recalcitrance, was significantly greater under CT83 than under NT83 (*P* < 0.05). This might be due to the more oxidative environment under CT as compared to NT management^59^. These results showed that C accumulation in coarse particles under NT was primarily in an easily decomposable form, a phenomenon which was observed for both long- and short-term NT. The current results were consistent with Bayer *et al*.^59^ who reported less humification of SOC under NT compared to CT in a soil where they examined the semi-quinone free radicals using electron spin resonance technique. Similar results were also reported by Milori *et al*.^60^, González-Pérez *et al*.^61^ and Martins *et al*.^16^ using the laser-induced fluorescence spectroscopy technique. Results from ^13^C NMR spectroscopy further showed that cultivation could significantly increase SOC mineralization and reduce the concentration of mineral-associated organic carbon^62^. Using solid-state ^13^C NMR technique, Ding *et al*.^51^ found less aliphatic and more aromatic carbon from soils under conventional compared to under conservation management.

**Table 4:** Impacts of tillage practices on distributions of aliphatic and aromatic C in soil particle size fractions.

Particle	Treatments^†^	Aliphatic C		Aromatic C		Aromatic/Aliphatic	
		Intensity	Integral	Intensity	Integral	Intensity	Integral
Sand	CT83	1.66b	278b	2.30a	320a	1.38a	1.15a
	CT97	1.84ab	300ab	2.08a	299a	1.13ab	0.99ab
	NT83	2.59a	399a	1.86a	277a	0.71b	0.69b
	NT97	2.11ab	338ab	1.89a	280a	0.90ab	0.83ab
Silt	CT83	1.13a	179a	3.66a	467a	3.23b	2.61a
	CT97	1.14a	196a	3.71a	460a	3.25b	2.35a
	NT83	0.85a	173a	4.03a	539a	4.74a	3.11a
	NT97	1.02a	167a	3.82a	496a	3.74ab	2.97a
Clay	CT83	1.54a	221a	4.03a	815a	2.61a	3.69a
	CT97	1.69a	233a	4.07a	806a	2.41a	3.46a
	NT83	1.86a	257a	4.57a	871a	2.46a	3.39a
	NT97	1.70a	240a	4.10a	842a	2.41a	3.50a

Values are means of four replicates.

^†^CT83, long-term conventional tillage established in 1983; CT97, short-term conventional tillage established in 1997 following 13 years of no-tillage (NT); NT83, long-term no-tillage established in 1983; NT97, short-term tillage established in 1997 following 13 years of CT.

^§^Different letters in the same column within each fraction indicate significant difference at 0.05 probability level.

In this study, the aromatic C in the silt-sized fraction was surprisingly higher under NT83 than under other treatments, indicating that long-term NT contributed to recalcitrant C storage in this size fraction. Aromatic/aliphatic C ratio was highest in the silt- as compared with other-sized fractions for all treatments. This is consistent with García-Oliva *et al*.^63^ who found that the silt-associated SOC had a slower depletion rate than other fractions when forest was converted to pasture. Saab and Martin-Neto^64^ also found that C was more stable in the silt- than in other-sized fractions of a Gleysol employing the electron paramagnetic resonance technique. The aromatic C was found dramatically higher in the clay- than in other-sized fractions for all treatments (Table 4). This agrees with the findings that the clay-sized fraction may preserve SOC from microbial attack by adsorbing SOC onto clay surfaces, by encapsulation of clay particles or entrapment of SOC in pores^65^. There were no significant differences (*P* > 0.05) of aliphatic and aromatic C in clay- and silt-sized fractions of NT, compared with CT soil (Table 4), which indicates that C in these fractions is not as sensitive to changes in land use management practices compared to the sand-size fractions^66^.

## Conclusions

The curve-fitting technique, used in combination with Fourier-transform mid-infrared spectra, can be a robust tool for determining the functional groups and structural features of soil organic carbon in soil particle size samples. Numerous overlapping absorption bands in MIR spectra of soil samples were enhanced and identified using this technique which advances our ability to qualitatively and semi-quantitatively analyze the spectrum and organic components of the samples. The curve-fitting analysis for mid-infrared spectra further indicates that tillage-induced differences in SOC characteristics were most obvious in sand-sized fraction with significant (i) greater aliphatic C content and (ii) smaller aromatic/aliphatic C ratio in long term NT compared to long-term CT.
